# Microbial Gut Diversity of Africanized and European Honey Bee Larval Instars

**DOI:** 10.1371/journal.pone.0072106

**Published:** 2013-08-21

**Authors:** Svjetlana Vojvodic, Sandra M. Rehan, Kirk E. Anderson

**Affiliations:** 1 Center for Insect Science, Department of Ecology and Evolutionary Biology, University of Arizona, Tucson, Arizona, United States of America; 2 Department of Biology, University of Pennsylvania, Philadelphia, Pennsylvania, United States of America; 3 United States Department of Agriculture, Tucson, Arizona, United States of America; Ghent University, Belgium

## Abstract

The first step in understanding gut microbial ecology is determining the presence and potential niche breadth of associated microbes. While the core gut bacteria of adult honey bees is becoming increasingly apparent, there is very little and inconsistent information concerning symbiotic bacterial communities in honey bee larvae. The larval gut is the target of highly pathogenic bacteria and fungi, highlighting the need to understand interactions between typical larval gut flora, nutrition and disease progression. Here we show that the larval gut is colonized by a handful of bacterial groups previously described from guts of adult honey bees or other pollinators. First and second larval instars contained almost exclusively Alpha 2.2, a core Acetobacteraceae, while later instars were dominated by one of two very different *Lactobacillus* spp., depending on the sampled site. Royal jelly inhibition assays revealed that of seven bacteria occurring in larvae, only one Neisseriaceae and one *Lactobacillus* sp. were inhibited. We found both core and environmentally vectored bacteria with putatively beneficial functions. Our results suggest that early inoculation by Acetobacteraceae may be important for microbial succession in larvae. This assay is a starting point for more sophisticated *in vitro* models of nutrition and disease resistance in honey bee larvae.

## Introduction

Insects are known to harbor microbial gut communities that provide protection from pathogens and contribute to nutrition. This is especially true for nutritionally limited organisms or those that subsist primarily on complex plant polymers [1]. Blood sucking insects and herbivorous insects in particular are believed to have coevolved with a number of bacterial and fungal symbionts that can aid in production of vitamins, nitrogen fixation and provide sterols [2]–[5]. In particular, acetic acid bacteria (AAB) are associated with insects that have sugar rich diets, and have been demonstrated to interact directly with the expression of antimicrobial peptides in the gut, affect larval development time, and contribute to cognitive function and general epithelial health [6]–[9]. *Lactobacillus* spp. in both solitary and social bees are thought to protect food stores and inhibit pathogenic microbes by lowering pH levels or producing secondary metabolites [10]–[12].

Results from non-cultured based sequencing indicate that adult honey bees have a distinct microbial gut community comprised of 7–12 core bacterial species belonging to the Acetobacteraceae, Betaprotobacteria, Gammaprotobacteria and Firmicutes [13]–[16]. While these studies focused primarily on the adult bee gut, non-cultured based studies of larvae have yielded minimal results. Direct PCR screening found that larvae were nearly devoid of putative core bacteria, with the exception of Alpha 2.2, an Acetobacteriaceae [14]. More extensive sampling found that last instar honey bee larvae harbored more diverse microbiota composed of both core and non-core bacteria [17]. Early culture-based work indicates that the minority of larvae contain microorganisms, suggesting that the presence of microbes in honey bee larvae is due to unwanted contamination [18], [19]. In general, microbial communities in the larval gut can differ dramatically from those of adults, revealing diverse groups of Gammaproteobacteria, Acetobacteraceae, Firmicutes, *Bacillus* spp., and various molds and yeast [17], [19], [20].

The nature of nutrients and their movement throughout the colony may be an important factor determining microbial abundance and diversity in larvae. The diet of the adult honey bee begins with nectar and pollen collection. Enzymes and microorganisms are added to the nectar increasing acidity, while the mechanical evaporation of water by worker bees generates unfavorable osmotic conditions for most microorganisms. Pollen is mixed with microbes, honey and/or nectar and ferments into beebread, a highly nutritious and microbially diverse food storage product [22]–[24]. Beebread is digested by young nurse bees, and converted to storage products in the hemolymph and fat body. Larvae are fed initially with nurse bee hypopharyngeal gland secretions (royal jelly), but these secretions are subsequently mixed with beebread, enzymatically active glandular material, and dilute honey (worker jelly).

Worker jelly is rich in protein, lipids, carbohydrates and micronutrients allowing the larvae to grow more than a hundred times the size of an egg in only 5 days. The ratio of royal jelly, pollen and sugar is not constant and jelly concentration decreases as bee larvae age [25]. Royal jelly is considered highly antimicrobial, possessing a pH between 3.6 and 4.2, and many peptides active against gram-positive and gram-negative bacteria, fungi and yeasts [26], [27]. Unlike the compartmentalized nature of the adult honey bee gut, developing larvae possess only a midgut, which connects with the hindgut at the pre-pupae stage [28]. Thus the larval midgut represents a unique niche for bacterial or fungal growth, and this stage of the honey bee life cycle is the target of many major pathogens including bacterial diseases European and American foulbrood, and fungal diseases stonebrood and chalkbrood [29]–[33].

Despite the recent literature on the microbiota of adult honey bees, currently there is very little and inconsistent information on the microbial communities in honey bee larvae. The aim of this study was to describe the larval gut microbiota from two apiaries: one with non-managed (Africanized) bees and the second with managed (European) bees. Since the new data using non-culture based techniques have shown limited or absent larval microbiota we chose to use traditional culture-based methodologies to isolate the gut microbiota of all five larval instars. Following characterization with 16S rDNA sequencing of the microbiota, we tested possible coadaptation of these bacteria with the larval host by surveying antimicrobial properties of royal jelly, a main component of honey bee larval food.

## Materials and Methods

### Bacterial Isolation

Honey bee larvae were collected from late April to mid May 2011. Three colonies were sampled at each of two sites. Site 1, Casa Grande apiary was located in Tucson, Arizona (32.26° N/111.00° S), and composed of managed Europeans bees with access to agriculture and ornamentals. Site 2, Page Ranch apiary, (32.60° N/110.88° S) had Africanized non-managed bees at a remote Sonoran desert site. Both collection sites are under the jurisdiction of the University of Arizona and the USDA Carl Hayden Bee Research Center has permission from the State of Arizona and the University of Arizona to sample and maintain honey bee colonies in these locations. Larvae of all 5 instars were collected on site using grafting tools or forceps, and stored in physiological saline (0.9% w/v NaCl, 0.1% w/v Tween 80, 0.1% w/v Peptone) for transport. Larvae were surface sterilized by rinsing them in 75% ethanol 3 times with a final wash in sterilized saline solution. We collected a total of 144 larvae from Site 1 and 202 larvae from Site 2. Larvae were sorted by the colony of origin and pooled based on their developmental stage (instar), which was determined by their body size. In order to extract gut microbiota we ground whole larvae in 500 µl of physiological saline. The content from each host colony/larval instar was streaked using the sterile microbial loop on 4 growth media: Man Rogosa Sharpe medium (MRS), Sabouraud Dextrose Agar (SDA), Potato Dextrose Agar (PDA), and ¼ strength *Bacillus* growth media (composition per liter: of 18 g agar, 2.5 g Yeast extract, 1 g Pancreatic digest of casein, pH 7.2). From Site 1 we used 3 Petri plates/growth media/instar, and for Site 2 we used 2 Petri plates/growth media/instar. Where appropriate we used the sterile wire to randomly transfer 3 colonies from each Petri dish plate to the individual liquid broth that corresponded to the agar media used on the Petri plates. Samples were incubated in the liquid media for 3 weeks. If growth was observed, a portion of the bacterial sample was taken out for 16S rDNA sequencing.

### DNA Extraction

We used a gram positive bacterial DNA extraction protocol (+Lysozyme). Prior to DNA isolation, isolates were pelleted from broth media by centrifugation. Samples were incubated at 37°C for 1 hour with 300 µL of Lysozyme Lysis Buffer (100 mM NaCl, 500 mM Tris [pH 8.0], Lysozyme 10 mg/mL). After incubation, 200 µL of SDS buffer (100 mM NaCl, 500 mM Tris [pH 8.0], 10% [wt./vol.] SDS) was added to the samples, incubated at 65°C for 10 min, then centrifuged at 12,000 g for 5 minutes. The samples were treated with 500 µL of Phenol, gently mixed, then centrifuged at 12,000 g for 5 minutes. The aqueous phase was transferred to a new tube containing 500 µL of Chloroform: Isoamyl alcohol (24∶1). The samples were gently mixed for 5 minutes, then centrifuged (12,000 g) for 5 min. The aqueous layer was transferred to a clean tube and DNA was precipitated by adding 0.5 volume ammonium acetate and 1 volume of isopropanol. After incubation at 0°C overnight, samples were pelleted by centrifugation at 12,000 g for 30 minutes and washed twice with 70% ethanol and once with 100% ethanol. DNA was pelleted, air dried, and resuspended in low Tris buffer. Bacterial 16S rRNA genes were amplified with the general bacterial primers 27F (5-AGAGTTTGATCMTGGCTCAG-3) and 1522R (5-AAGGAGGTGATCCANCCRCA-3) [35]. The PCR program was 9 min at 95°C, followed by 15 cycles of 1 min at 95°C, 1 min at 55°C, and 2 min at 72°C; and a final extension step of 60°C for 10 min.

### Sequence Analyses

Chromatograms were visually inspected and sequences were bidirectionally aligned in BioEdit [35]. Multiple sequence alignments were performed in MAFFT [36] and Clustal [37]. Sequences were all trimmed to 603 base pairs of 16S ribosomal DNA with no missing bases. 16S alignments were clustered into operational taxonomic units (OTUs) using a 0.97 sequence similarity cutoff for designating phylotypes. OTUs were compared to GenBank with BLAST to identify their top hit. All sequences have been deposited in GenBank under accession numbers JX896451 - JX896641. Maximum parsimony (MP) analyses were conducted using PAUP* b4.10 [36]. One hundred random sequence stepwise additions were used in the MP analysis, holding 10 trees at each step and with tree bisection and reconnection for searching tree space. Node support was estimated using 500 bootstrap pseudoreplicates, using the same methods as for the heuristic search, and retaining compatible groups with less than 50% bootstrap support. Monophyletic haplotypes were identified by comparing each sample sequence to the GenBank reference library using BLAST (http://blast.ncbi.nlm.nih.gov/Blast.cgi).

### Inhibition Assays

Inhibiting properties of royal jelly were tested on bacterial isolates from each of the eight clades. Three bacterial strains were randomly selected from each clade and grown on three Petri dish plates in the presence of 5 filter paper discs containing commercial royal jelly. The strains tested were: Acetobacteraceae (strains CS1, CS5, AP14); *Bifidobacterium* (strains A11, A15, A30), Neisseriaceae (strains AB10, AS2, A17); *Fructobacillus fructosus* (strains A55, A60, A58); *Lactobacillus kunkeei* (strains CG43, CG37, CG74); *Lactobacillus* sp. A (strains A45, A29, A21), *Lactobacillus* sp. B (strains CG3, A101, A37) and *Bacillus* sp. (strains SP10). As a control bacteria we used *Staphylococcus* sp. (strain 3049 isolated from *Acacia* flower), *Streptomyces* spp. (strains 3377, 3375, 3373 isolated from bee bread) and *L. kunkeei* (strains 3077, 3076 isolated from *Acacia* flower). Control bacteria were isolated and frozen in July 2012. Bacterial isolates were transferred from the stock broth by pipetting 100 µl of the broth on to the agar plates. Each bacterial isolate was evenly spread with a Drigalski spatula on 3 Petri dish plates with 25 mL MRS, SDA or PDA agar depending on the original growth media used. Broth was allowed to dry for a few minutes before 5 sterile filter paper disks with the commercial royal jelly were placed on each agar plate, resulting in a total of 3 plates per strain, totaling 15 royal jelly disks per bacterial clade. The agar plates were then incubated in an anaerobic incubator at 34°C for 3–7 days depending on the speed of bacterial growth. We visually assessed and measured the bacterial growth diameter directly next to the royal jelly disk and in the area between the disks. Prior to use, the royal jelly was tested for bacterial presence and no bacterial growth was observed. No inhibition of bacteria was classified as such if the bacterial colonies were observed next to the filter paper with the royal jelly. Bacterial growth was enhanced by the royal jelly presence if the bacterial density was higher around the royal jelly disc in comparisons to the size and number of bacterial colonies observed between the royal jelly discs.

## Results

### Species of Bacteria Found in each Site

**Figure 1:**
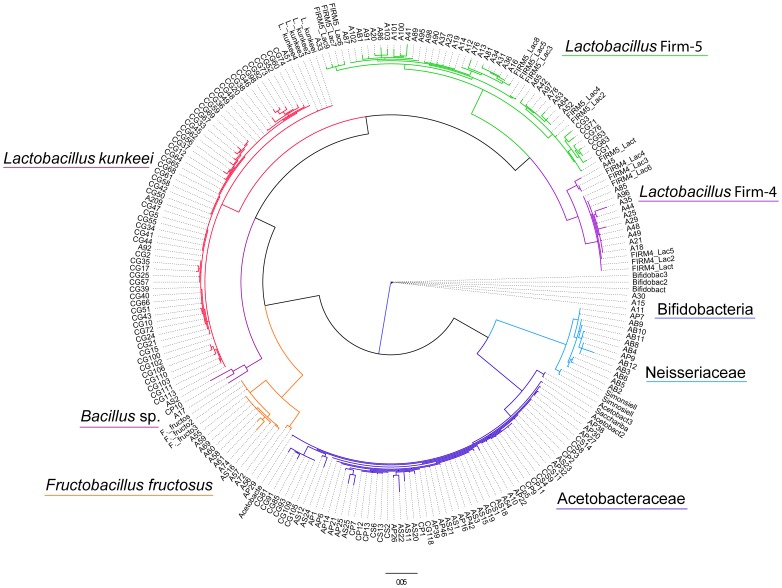
Maximum-parsimony consensus tree containing 186 bacterial strains isolated from honey bee larvae and 31 reference strains taken from previous publications identifying adult bee gut bacteria (see File S1). Bootstrap values over 50% are shown.

A total of 186 isolates were compared based on their 16S rRNA gene sequences and classified as the following bacterial phylotypes: a) Alphaproteobacteria, acid forming gram-negative bacteria; b) Betaproteobacteria belonging to family Neisseriaceae, gram-negative bacteria; c) Firmicutes, gram-positive bacteria belonging to genera *Bacillus* and *Lactobacillus,* and genus *Fructobacillus* formally classified in genus *Leuconostoc*; and d) Actinobacteria, genus *Bifidobacterium,* also acid forming gram-positive bacteria (Fig. 1). We did not find any species from the class Gammaproteobacteria that were previously found in adults and larvae of honey bees [15], [17]. Bacterial isolates in our phylogenetic tree are consistent with multiple studies of adult honey bees, demonstrated by incorporating several published clone and isolate 16S rDNA sequences in our phylogenetic tree (see File S1 for GenBank sequence accession numbers). The majority of Alphaproteobacteria strains were 99% similar to the strains isolated from honey bees by Mohr and Tebbe [17] with few new strains such as AS22 with only 90% similarity. Betaproteobacteria isolates were 96–98% similar to the strains previously isolated from adult honey bees [39]. The isolates from the Firmicutes group: *L. kunkeei* were 99% similar to isolates that were described from honey bees by Neveling et al. [40]; *Lactobacillus* sp. A and B were 98–99% similar to bacterial strains previously described by Martinson et al. [15], while *F. fructosus* strain AP29 strain was 97% similarity to *Bacillus* spp. described by Martinson et al. [15], and A60 strain was 99% similar *F. fructosus*. *Bifidobacterium* isolates were 99% similar to isolates described by Vásquez et al. [41] and Olofsson et al. [42]. Few *Bacillus* isolates were 99% similar to *Bacillus subtilis* [43] and 99% to *Bacillus megaterium*.

### Bacterial Diversity

**Figure 2:**
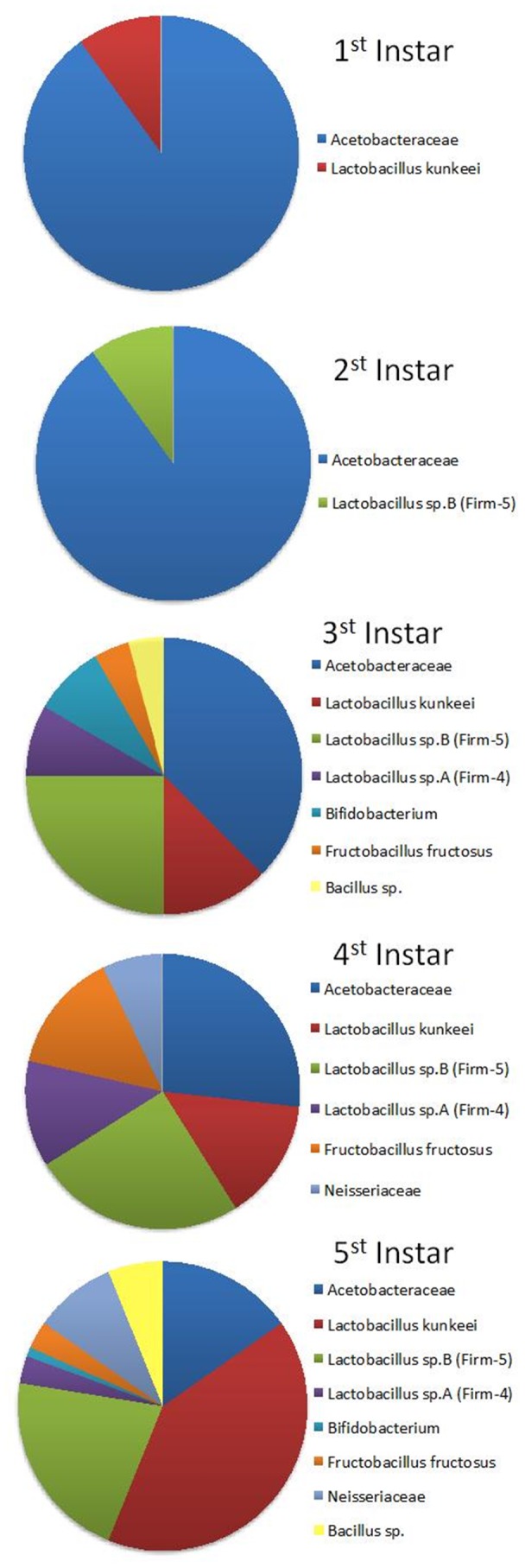
Combined bacterial phylotypes isolated from both European and Africanized apiaries and five honey bee larval instars.

We did not directly quantify each clade, and the relative abundance of bacterial clades presented here depends on the random amplification of bacteria growing on the medium. The most dominant isolates were from lactic acid forming bacteria in genus *Lactobacillus* (63%) of which 28% of isolates were classified as *L. kunkeei*, and the remaining *Lactobacillus* species were classified into two distinct clusters *Lactobacillus* sp. A also know as Firm-4 (6.4%) and *Lactobacillus* sp. B (Firm-5) (23%). The second most common isolate (27%) was Acetobacteraceae, corresponding to Alpha 2.2 [14]. *Fructobacillus* and Neisseriaceae each comprised 6% of all isolates; and least frequent were isolates from genera *Bacillus* (1.6%) and *Bifidobacterium* (1.6%) (Fig. 2).

**Figure 3:**
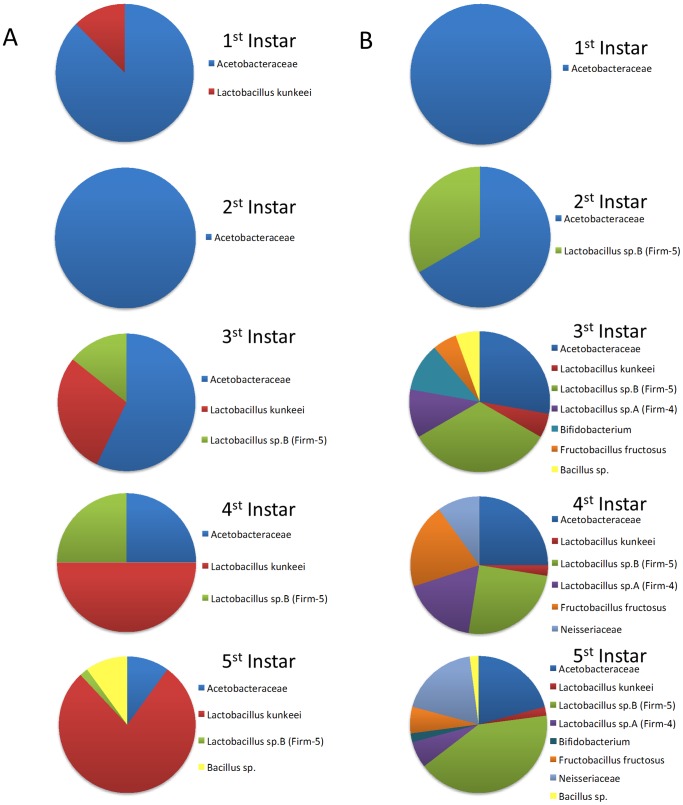
Bacterial phylotypes isolated from different larval instars and two different locations: (A) Casa Grande apiary with managed European bees; (B) Page Ranch apiary with non-managed Africanized bees.

There was variation between the two collection sites. At Site 1 (Casa Grande) 81 isolates were sequenced and only three bacterial lineages were found. This site was dominated by *L. kunkeei* (63%), Acetobacteraceae (30%), and *Lactobacillus* sp. B (7.5%). All six bacterial clades were found at Site 2 (Page Ranch) from 107 sequenced bacterial cultures. The most common isolates in Site 2 were *Lactobacillus* sp. B (35%) and Acetobacteraceae (25%). Somewhat less common were *Fructobacillus fructosus* (11%), *Lactobacillus* sp. A (10%) and Neisseriaceae (10%). In contrast to Site 1, *L. kunkeei* was the least abundant (2%) together with *Bacillus* isolates (2%) (Fig. 3).

### Bacterial Succession Through Honey Bee Larval Instars

Bacterial diversity increased from first instar to older larvae as expected due to their closed gut anatomy. First and second larval instars contained Acetobacteraceae, *L. kunkeei* and *Lactobacillus* sp. B. Fifth instar larvae had up to 7 bacterial clades. As mentioned above, most of the diversity was observed in non-managed Africanized larvae collected from Page Ranch (Site 2) and only Acetobacteraceae, *Bacillus sp*., *L. kunkeei* and *Lactobacillus* sp. B were present in managed Europeans bees (Site 1). In fifth instar larvae from managed European colonies the most abundant isolate was *L. kunkeei*, but in fifth instar larvae of Africanized bees, this bacteria was almost entirely absent and the most dominant bacteria was from a separate clade, *Lactobacillus* sp. B. Acetobacteraceae sp. was in similar abundance in both collection sites (Fig. 3).

### Culturing Media

*Lactobacillus kunkeei*, *Lactobacillus* sp. A and B, *Fructobacillus fructosus*, and *Bifidobacterium* sp. were most successfully cultured on Man Rogosa Sharpe medium (MRS). Acetobacteraceae and *Bacillus* were effectively cultured on: Sabouraud Dextrose Agar (SDA), Potato Dextrose Agar (PDA), and MRS. Neisseriaceae isolates were cultured on ¼ strength *Bacillus* growth media, but one strain was also successfully isolated from PDA media.

### Inhibition Test

**Figure 4:**
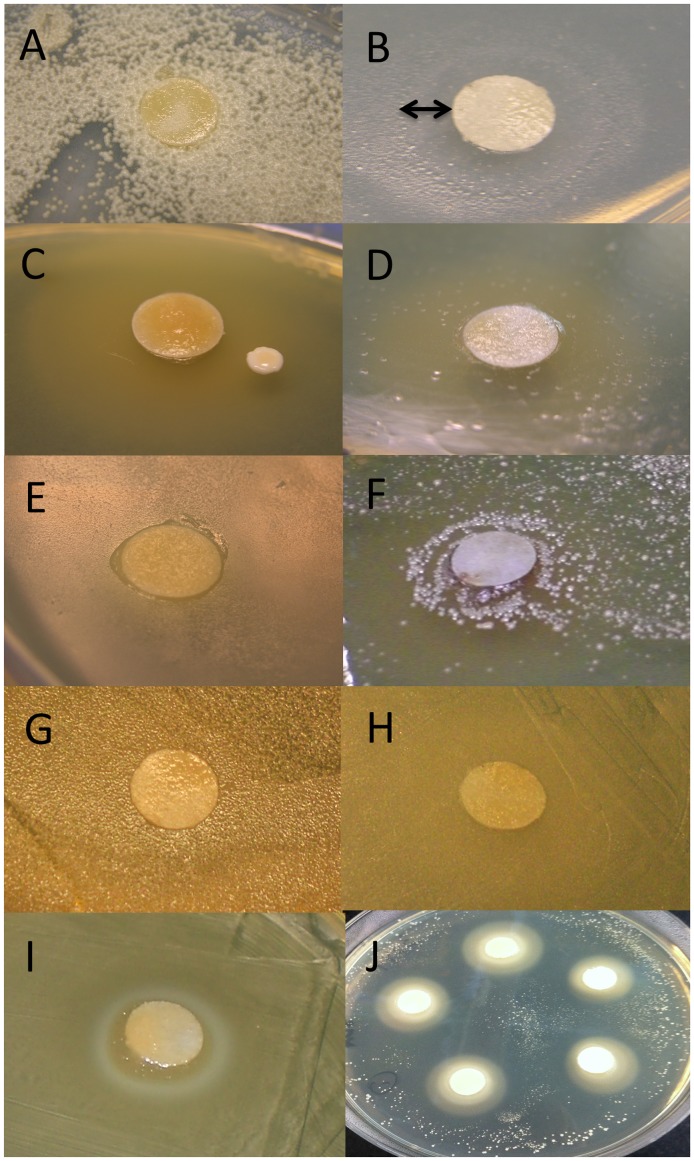
An example of honey bee larval gut bacteria from each identified phylotype and observed growing next to filter paper disks covered in royal jelly: (A) Acetobacteraceae; (B) Neisseriaceae; (C) *Lactobacillus* sp. B; (D) *Bifidobacterium*; (E) *Bacillus* sp.; (F) *Fructobacillus fructosus*; (G) *Lactobacillus kunkeei*; (H) *Lactobacillus* sp. A. Both control bacteria were inhibited (I) *Lactobacillus kunkeei* isolated from a flower; (J) *Staphylococcus sp.* isolated from a bee-bread (pollen stored in honeycomb cells). Some reduction in growth around royal jelly disc was recorded for Neisseriaceae as shown by the arrow.

Some inhibition of bacterial growth by royal jelly was observed for the strains of Neisseriaceae and very weak inhibition of *Lactobacillus* sp. A (Fig. 4). The rest of the phylotypes were not inhibited and in the case of Acetobacteraceae and *Fructobacillus fructosus* the density of bacterial colonies was higher around the royal jelly disk. Control bacteria were *Staphylococcus* sp. and *L. kunkeei* spp. isolated from *Acacia* flowers, and *Streptomyces* spp. isolated from bee bread. *Staphylococcus* spp. were highly inhibited, while *Streptomyces* spp. growth was not affected (Table 1). Interestingly we observed variation in *L. kunkeei* inhibition based on the strain origin; larval gut strains tested were not inhibited and strains isolated from flowers were inhibited by royal jelly.

**Table 1:** Inhibition test of bacteria by pure royal jelly on agar plates.

Bacteria	Mean zone of inhibition ± SE
**Control**	
* Staphylococcus* sp. (strain 3049) (flowers)	17.5±1
* Lactobacillus kunkee*i (strains 3077, 3376) (flowers)	6.9±0.4
* Streptomyces* sp. (3377, 3375, 3373) (bee bread)	0.6±0.2
**Bacteria from the larval gut**	–
Acetobacteraceae (strains CS1, CS5, AP14)	–
Neisseriaceae (strains AB10, AS2, A17)	2.9±1
* Lactobacillus* sp. B (strains CG3, A101, A37)	–
* Bifidobacterium* (strains A11, A15, A30)	–
* Bacillus* sp. (strains SP10)	–
* Fructobacillus fructosus* (strains A55, A60, A58)	–
* Lactobacillus kunkeei* (strains CG43, CG37, CG74)	–
* Lactobacillus* sp. A (strains A45, A29, A21)	1.5±0.7

Bacterial samples were isolated from honey bee larvae and the control bacteria were isolated from flowers and bee bread. Mean diameter of zones of inhibition (in millimetres ± S.E.).

**−** = No zone of inhibition observed.

## Discussion

Consistent with the results of recent culture independent studies we demonstrated via different growth media that honey bee larvae harbor a subset of gut microbiota that is similar to the core microbiota found previously in adult honey bees. Relative diversity of different bacterial clades were not consistent between the two collection sites, suggesting that at least some part of the gut microbial community is dependent on the bee species (social organism) or the environment. Consistent with the findings of Evans and Armstrong [20] there was an increase in the number of isolated bacterial colonies and diversity as the larvae aged. One possible explanation could be due to the larval gut environment and the absence of defecation during the larval stage, as well as change in diet as larvae become older. First and second larval instars had at most three bacterial colonies per Petri dish plate, while plates from older larvae were covered with hundreds of bacterial colonies. This suggests that larvae might be contaminated with some of the bacterial strains very early on, but these bacteria were able to reproduce and thrive within the larval gut in spite of the antimicrobial properties of royal jelly.

Furthermore, Neisseriaceae and *Lactobacillus* sp. A growth was slightly inhibited due to the presence of royal jelly on the plate, while the rest of the clades were unaffected or their growth was enhanced. We also demonstrated that different species or strains of bacteria originating from flowers were inhibited by the royal jelly such as *Staphylococcus* and *L. kunkeei*. While *L. kunkeei* were found in both larval guts and flowers, their inhibition was only observed for the strains isolated from flowers. This finding demonstrates an interesting adaptation among the same bacterial species, possibly due to differences in growing environment (flower vs. honey bee hive or gut). This observation warrants further research into strain diversity and the functional genomics of *L. kunkeei* strains. Although royal jelly possesses antimicrobial peptides, proteins and flavonoids that can inhibit the growth of some common bacterial pathogens [44]–[48], it was previously hypothesized by Anderson et al. [49], and here demonstrated, that bacteria adapted to acidic gut environments can use dilute or concentrated royal jelly as a growth medium.

Adult honey bees have three basic food sources: antiseptic royal jelly, honey, and beebread; all microbially diverse food storage products [21]. Therefore, we suggest that the three most common bacteria found in larvae, Acetobacteraceae, *L. kunkeei*, and *Lactobacillus* sp. B, can be “rejuvenated” from honey and/or beebread [50]–[56], suggesting that the food stores may be one source of larval inoculum. While Acetobacteraceae, *Bifidobacterium*, *Lactobacillus* sp. A and *Lactobacillus* sp. B are considered part of the core adult bee microbiota, *L. kunkeei* and *Fructobacillus* are potentially vectored from nectar sources [53] and have been found with sporadic abundance in many different locations and pollinators, possibly due to environmental fluctuations [12], [15], [51], [57]–[60]. These two bacteria have not emerged as part of the core gut microbiota of adult honey bees, and are undetected or found in minuscule proportions in non-culture based approaches [13]–[17], [57], [61], [62].

Although found at lowest frequency, bacteria cultured from the guts of first and second instar larva may be the most biologically significant and critical for the course of bacterial succession [63]. Although strains of Acetobacteraceae are considered part of the core adult microbiota, they also were one of the few bacteria cultured from 1^st^ and 2^nd^ instar larvae. Acetobacteraceae (Alpha 2.2) is highly aerotolerant (Anderson unpublished data), and is found at low frequency in the adult midgut, but does not occur in the increasingly anoxic adult hindgut [14]. Bacteria from this clade occur in both larvae and nurse bees suggesting that Alpha 2.2 may be best adapted to food stores and the larval gut, and/or associated with nurse bee hypopharyngeal glands. Acetobacteraceae found in larvae are similar in 16S sequence to bacteria occurring in floral pollen and the pollen provisions of both solitary and social bees, suggesting a recent or enduring association with the floral niche [12], [15], [64]. That the growth of Alpha 2.2 is actually enhanced in the presence of harsh antimicrobial compounds found in royal jelly further suggests a long association with honey bee larvae, or some degree of pre-selection in another specialized antimicrobial niche like nectar.

Besides Acetobacteraceae, later larval instars were also colonized by *Fructobacillus* but only at Site 2, while *L. kunkeei* was dominant at Site 1 and was scarce at the other. Interestingly in controlled foraging studies, both *Fructobacillus* and *L. kunkeei* were abundant in free-flying honey bees, but remained undetected in honey bees denied access to the pollination environment [57]. Possibly the concentration of these bacteria in favorable portions of the gut and food stores varies with an environmental factor like nectar type or relative humidity. Differences in diversity among sites may reflect a competitive advantage held by *L. kunkeei* as opposed to management practices or host genotype. The core gut bacteria have co-evolved as a community, but when present in large numbers, *L. kunkeei* may temporarily dominate many of the behaviorally mediated niches of the honey bee hive including beebread and the larval gut. It may be that *L. kunkeei* and to a lesser degree, *Fructobacillus* play minor roles in the microbial community of floral nectar, but can experience competitive release in the hive environment under favorable conditions.

Nurse bees consume beebread composed of partially processed pollen that is converted to royal jelly by the hypopharyngeal glands. Royal jelly is then fed to the queen larvae and early worker instar larvae. In later instars, royal jelly is mixed with honey/nectar and pollen [25]. It is unknown if pollen is incidental to the larval feeding process, but pollen grains are digested by larval guts, and suspected to contribute 10% of larval nutrition [65]. The duct of the hypopharyngeal gland exits at the mouth, decreasing the chance of microbial contamination as royal jelly is transferred to early stage larvae. That very few pollen grains [65] or bacteria can be found in the midguts of first instar larvae, is consistent with little bacterial inoculation from pollen or beebread. However, when these glandular secretions are mixed with increasing amounts of sugary crop contents, the path traveled by the larval food is more susceptible to microbial inoculation from honey, the crop and beebread. Our study and others suggest that both bacteria and pollen become more abundant in later instar larvae [66] contributing to increased diversity and abundance with successive larval instars.

Although pollen appears to be an incidental part of the honey bee larval diet, pollen digestion is an ancestral trait. The larvae of solitary ancestors develop by feeding directly on carefully measured pollen balls buried in the soil or in a tree cavity. Like honey bee beebread, the pollen provisions of solitary bees are infused with concentrated nectar, and can harbor Acetobacteraceae and *L. kunkeei* [12]. The fermentative action of these bacteria is suspected to decrease the chance of larval contamination and preserve the nutritive value of pollen for larval development. Similar to larval diseases of the honey bee, species of yeasts and filamentous fungi are primary pathogens of solitary bee larvae, and can often decimate a significant fraction of the population [65]. Collectively, this suggests that the spatial separation of food stores from developing larvae in many social bees was due to selection by microbial pathogens. Among other things, this key innovation in social bee evolution has allowed the microbial filtering of larval food stores, perhaps limiting the contamination of early instars with pathogens lurking in beebread. One might imagine an incipient honey bee hypopharyngeal gland contributing to parental care by enhancing the antimicrobial character of pollen provisions via the generation of an enzyme like glucose oxidase.

## Conclusions

Inferring the source of larval inoculation requires an understanding of bacterial communities typically harbored by, or potentially contaminating larval food provided by nurse bees. Very few bacteria can deal with the osmotic stress and the acidity associated with high concentration of sugars subsequently added to royal jelly. Associated with the shift from royal to worker jelly, bacterial diversity increased probably due to the increase in pollen in the larval food. As the pollen grains becomes mixed with dilute honey and make their way into the gut of later instar larvae there is an incredible jump in pH from acidic to neutral, suggesting that many acidophiles adapted to nectar, beebread and honey may have an ephemeral existence in the larval gut. Even so, larval microbiota does not dramatically differ from the adult bee gut microbiota. While these bacteria may play a role in nutrient processing in adult bees, they might contribute to larval immunity during the early and fragile stage of honey bee development.

## Supporting Information

File S1 **The list of 31 bacterial reference strains taken from previous publications that were incorporated in** **Figure 1** **.**
(DOCX)
