# Supplementary material for: Microbial Gut Diversity of Africanized and European Honey Bee Larval Instars
Source: PLoS One. 2013 Aug 21;8(8):e72106. doi: 10.1371/journal.pone.0072106 (PMC3749107; doi:10.1371/journal.pone.0072106)
Supplement: File S1 — The list of 31 bacterial reference strains taken from previous publications that were incorporated in Figure 1 . (DOCX) [file pone.0072106.s001.docx]

FIRM-4 *Lactobacillus* Cox-Foster gi|111143428|gb|DQ837633.1|

FIRM-4 *Lactobacillus* Cox-Foster gi|111143427|gb|DQ837632.1|

FIRM-4 *Lacto*bacillus Martinson gi|298397348|gb|HM112042.1|

FIRM-4 *Lactobacillus* Bin4 Olofsson and Vasquez gi|145652877|gb|EF187245.1|

FIRM-4 *Lactobacillus* Hon2 Olofsson and Vasquez gi|145652876|gb|EF187244.1|

FIRM-4 *Lactobacillus* Martinson gi|111143427|gb|DQ837632.1|

FIRM-5 *Lactobacillus* Cox-Foster gi|111143429|gb|DQ837634.1|

FIRM-5 *Lactobacillus* Cox-Foster gi|111143430|gb|DQ837635.1|

FIRM-5 *Lactobacillus* Cox-Foster gi|111143431|gb|DQ837636.1|

FIRM5 *Lactobacillus* Martinson gi|298397339|gb|HM112033.1|

FIRM-5 *Lactobacillus* Martinson gi|298397186|gb|HM111880.1|

FIRM-5 *Lactobacillus* Cox-Foster gi|111143432|gb|DQ837637.1|

FIRM5 *Lactobacillus* Cox-Foster gi|38224750|gb|AY370183.1|

FIRM-5 *Lactobacillus* Martinson gi|298398466|gb|HM113160.1|

FIRM-5 *Lactobacillus* Martinson gi|298397410|gb|HM112104.1|

*F. fructosus* Martinson gi|298397946|gb|HM112640.1| Arizona, Host: Diadasia opuntiae (Adult gut)

*F. fructosus* He et al. gi|307557177|gb|HQ180388.1| China, Host: Camponotus japonicus (Adult gut)

*F. fructosus* Koch and Schmid-Hempel gi|305430523|gb|HM215039.1| Central Europe, Host: bumblebee (Adult gut)

*L. kunkeei* Edwards et al. gi|219846812|ref|NR_026404.1|California, grape juice

*L. kunkeei* Martinson gi|298397891|gb|HM112585.1| Arizona, Host: *Diadasia opuntiae* (Adult gut)

*L. kunkeei* Olofsson and Vasquez gi|145652871|gb|EF187239.1|Sweden, honey bee (Adult crop)

*L. kunkeei* Tajabadi et al. gi|256682124|gb|GQ451618.1|Malaysia, *A. cerana* (Adult crop)

Acetobacteriaceae Morr and Tebbe gi|66766069|emb|AJ971850.1| Germany, honey bee (Adult gut)

Acetobacteriaceae Martinson gi|298393792|gb|HM108486.1| Malaysia, *A. dorsata* (adult gut)

Saccharibacter sp. Jojima et al. gi|219857191|ref|NR_024819.1| Japan, Pollen

Acetobacteriaceae Martinson gi|298397574|gb|HM112268.1|Arizona, *Xylocopa californica* (adult gut)

*Simnosiella* sp. Jeyaprakash et al. gi|38224756|gb|AY370189.1|Florida, honey bee (adult ground up tissue)

*Simonsiella* sp. Disayathanoowat et al. gi|295393236|gb|HM008719.1| Thailand, honey bee (adult midgut)

*Bifidobacterium* Jeyaprakash et al. gi|38224751|gb|AY370184.1| Florida, honey bee (adult ground up tissue)

*Bifidobacterium* Olofsson and Vasquez gi|145652866|gb|EF187234.1| Sweden honey bee (Adult crop)

*Bifidobacterium* Martinson et al. gi|298398596|gb|HM113290.1|Arizona, Honey bee (Adult gut)

References:

1. Olofsson TC, Vasquez A (2008) Detection and identiﬁcation of a novel lactic acid bacterial ﬂora within the honeybee *Apis mellifera*. Curr Microbiol 57: 356-363.
2. He H, Chen Y, Zhang Y, Wei C (2011) Bacteria associated with gut lumen of *Camponotus japonicus* Mayr. Environ Entomol 40: 1405-1409.
3. Cox-Foster DL, Conlan S, Holmes EC, et al. (2007) A metagenomic survey of microbes in honey bee colony collapse disorder. Science 318: 283-287.
4. Martinson VG, Danforth BN, Minckley RL, Rueppell O, Tingek S, Moran N (2011) A simple and distinctive microbiota associated with honey bees and bumble bees. Mol Ecol 20: 619-628.
5. Edwards CG, Haag KM, Collins MD, Hutson RA, Huang YC (1998) *Lactobacillus kunkeei* sp. nov.: a spoilage organism associated with grape juice fermentations. J Appl Microbiol 84: 698-702.
6. Tajabadi N, Mardan M, Abdul Manap MY et al. (2011) Detection and identification of Lactobacillus bacteria found in the honey stomach of the giant honeybee Apis dorsata. Apidologie, 42, 642–649.
7. Mohr,K.I. and Tebbe,C.C. 2007. Field study results on the probability and risk of a horizontal gene transfer from transgenic herbicide-resistant oilseed rape pollen to gut bacteria of bees. Appl. Microbiol. Biotechnol. 75 (3), 573-582.
8. Jojima,Y., Mihara,Y., Suzuki,S., Yokozeki,K., Yamanaka,S. and Fudou,R. 2004. Saccharibacter floricola gen. nov., sp. nov., a novel osmophilic acetic acid bacterium isolated from pollen. Int. J. Syst. Evol. Microbiol. 54 (PT 6), 2263-2267.
9. Jeyaprakash,A., Hoy,M.A. and Allsopp,M.H. 2003. Bacterial diversity in worker adults of Apis mellifera capensis and Apis mellifera scutellata (Insecta: Hymenoptera) assessed using 16SrRNA sequences. J. Invertebr. Pathol. 84 (2), 96-103.
10. Disayathanoowat,T., Young,J.P., Helgason,T. and Chantawannakul,P. 2012. T-RFLP analysis of bacterial communities in the midguts of Apis mellifera and Apis cerana honey bees in Thailand. FEMS Microbiol. Ecol. 79 (2), 273-281.
